# Supplementary figures and images for: Disulfiram and copper combination therapy targets NPL4, cancer stem cells and extends survival in a medulloblastoma model
Source: PLoS One. 2021 Nov 3;16(11):e0251957. doi: 10.1371/journal.pone.0251957 (PMC8565761; doi:10.1371/journal.pone.0251957)

Supplementary Figure 1

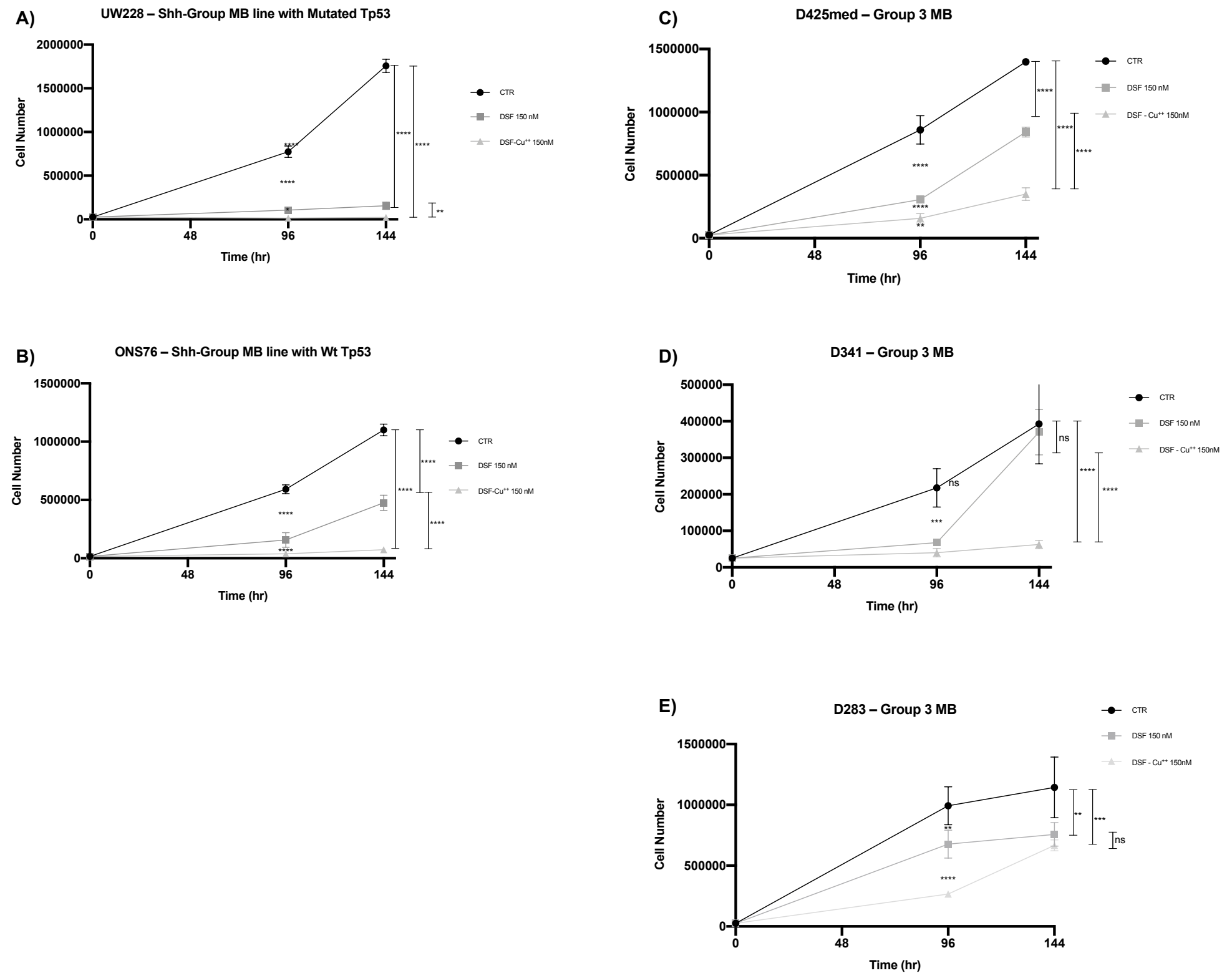

Supplement: S1 Fig — Combination of DSF and Cu++ determines a significant reduction in proliferative rate in all the lines when compared to untreated controls and DSF-treated cells. Cells were treated after 24h from plating with DSF or DSF-Cu++ 150nM and counted at 96h and 144h post-treatment. (PDF) [file pone.0251957.s001.pdf]

Supplementary Figure 2

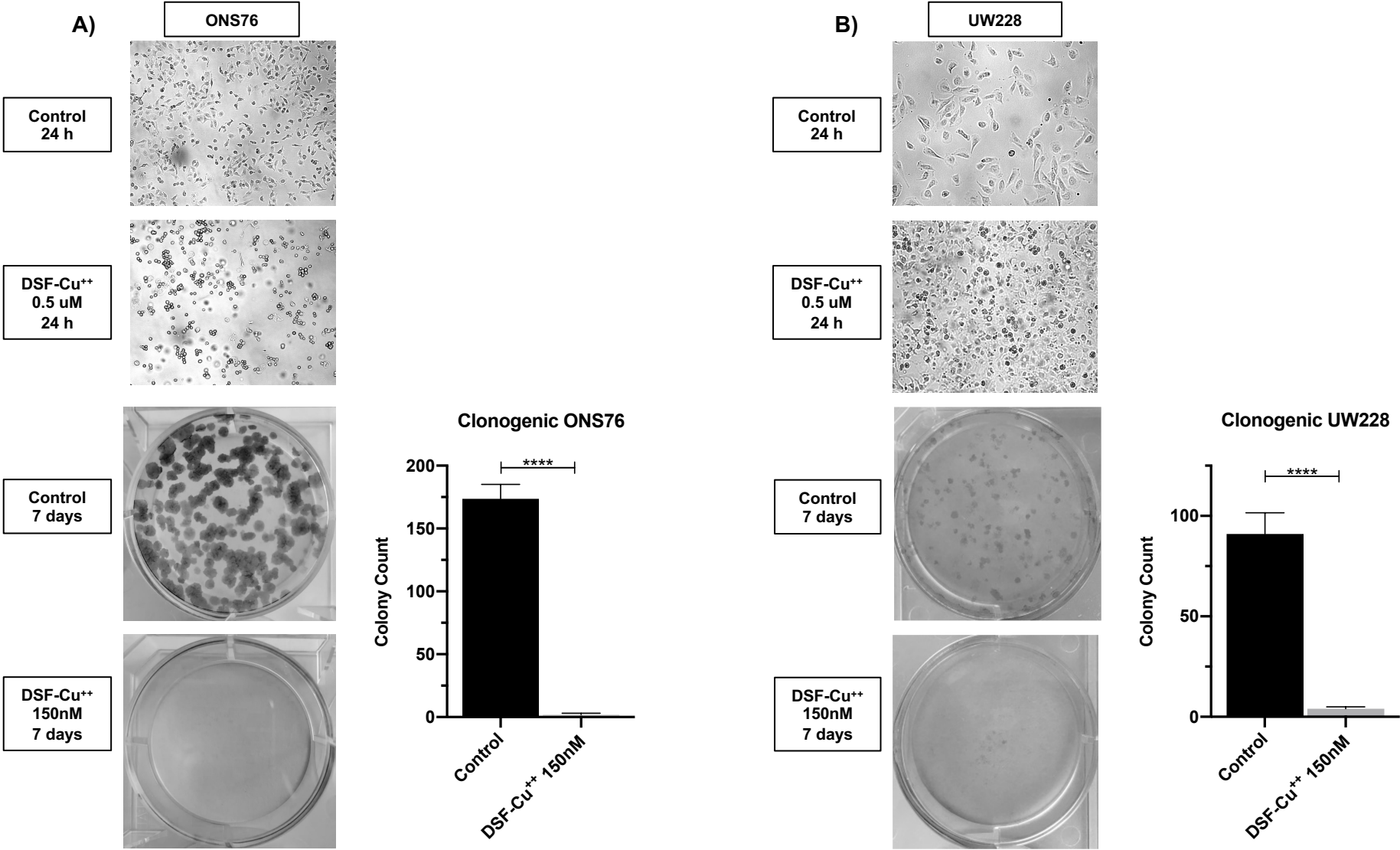

Supplement: S2 Fig — ONS76 and UW228 clonogenic potential is significantly reduced by 7 days of combination therapy with DSF-Cu++ 150nM. (PDF) [file pone.0251957.s002.pdf]

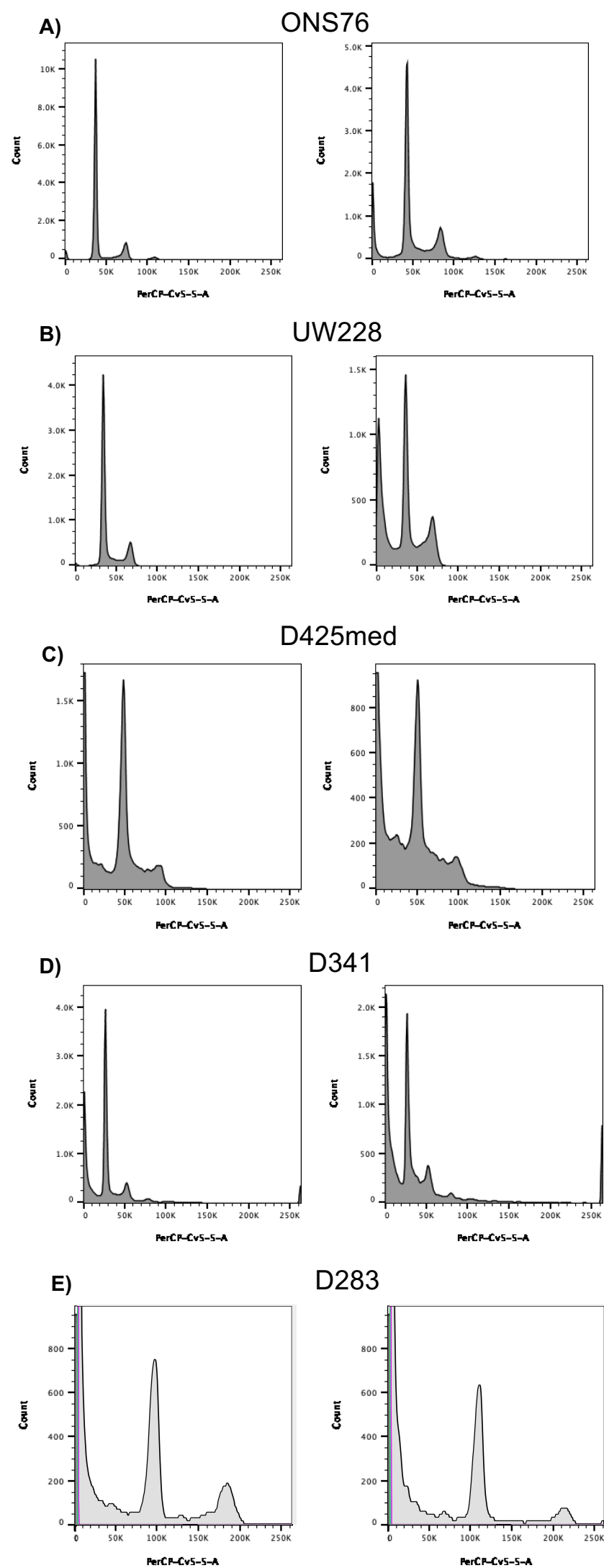

Supplement: S5 Fig — Cell cycle analysis through PI staining of DNA content was carried out in ONS76, UW228, D425med and D341 cell-lines. An increase in sub-G0/G1 cells was noticed after 48 hours of treatment with 150nM DSF-Cu++, consistently with AnnV/PI data. (PDF) [file pone.0251957.s005.pdf]

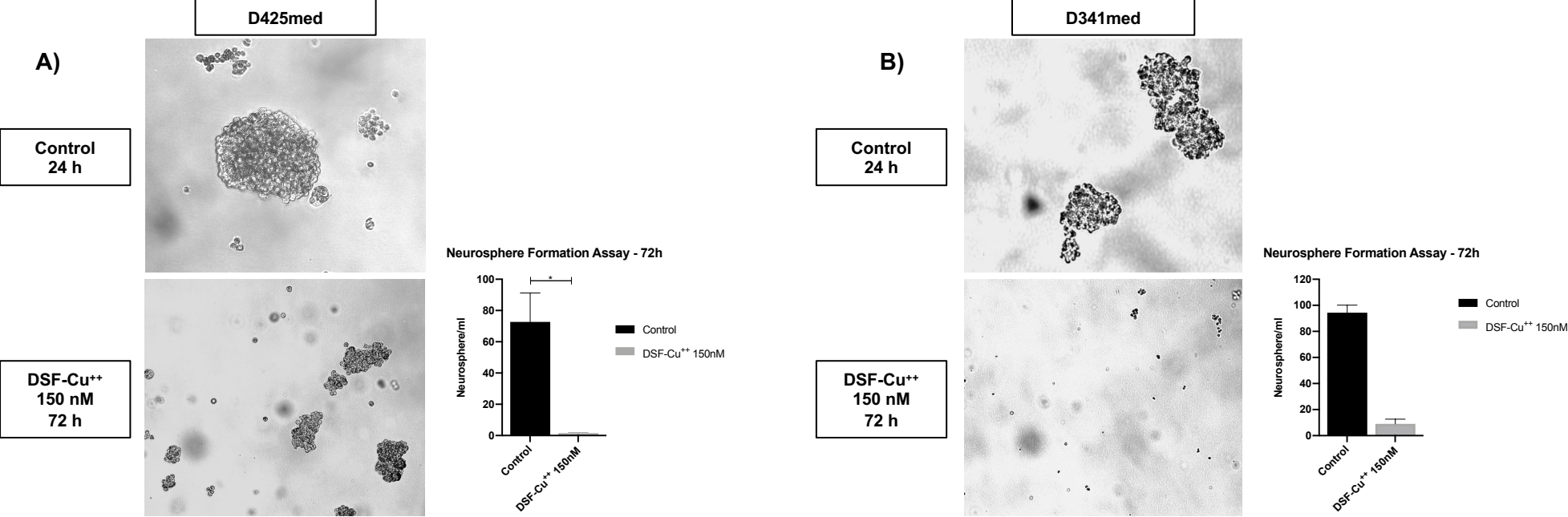

Supplementary Figure 6

Supplement: S6 Fig — Neurosphere formation was greatly reduced after 72 hours of treatment with 150nM DSF-Cu++ in D425med and D341 cells. High-Dose DSF-Cu++ reduces ALDH+ cancer cells in D425med and D341 lines. 2uM DSF-Cu++ induces significant reduction in ALDH+ cells in D425med and D341 after 2 hours of treatment. (PDF) [file pone.0251957.s006.pdf]

Panel A

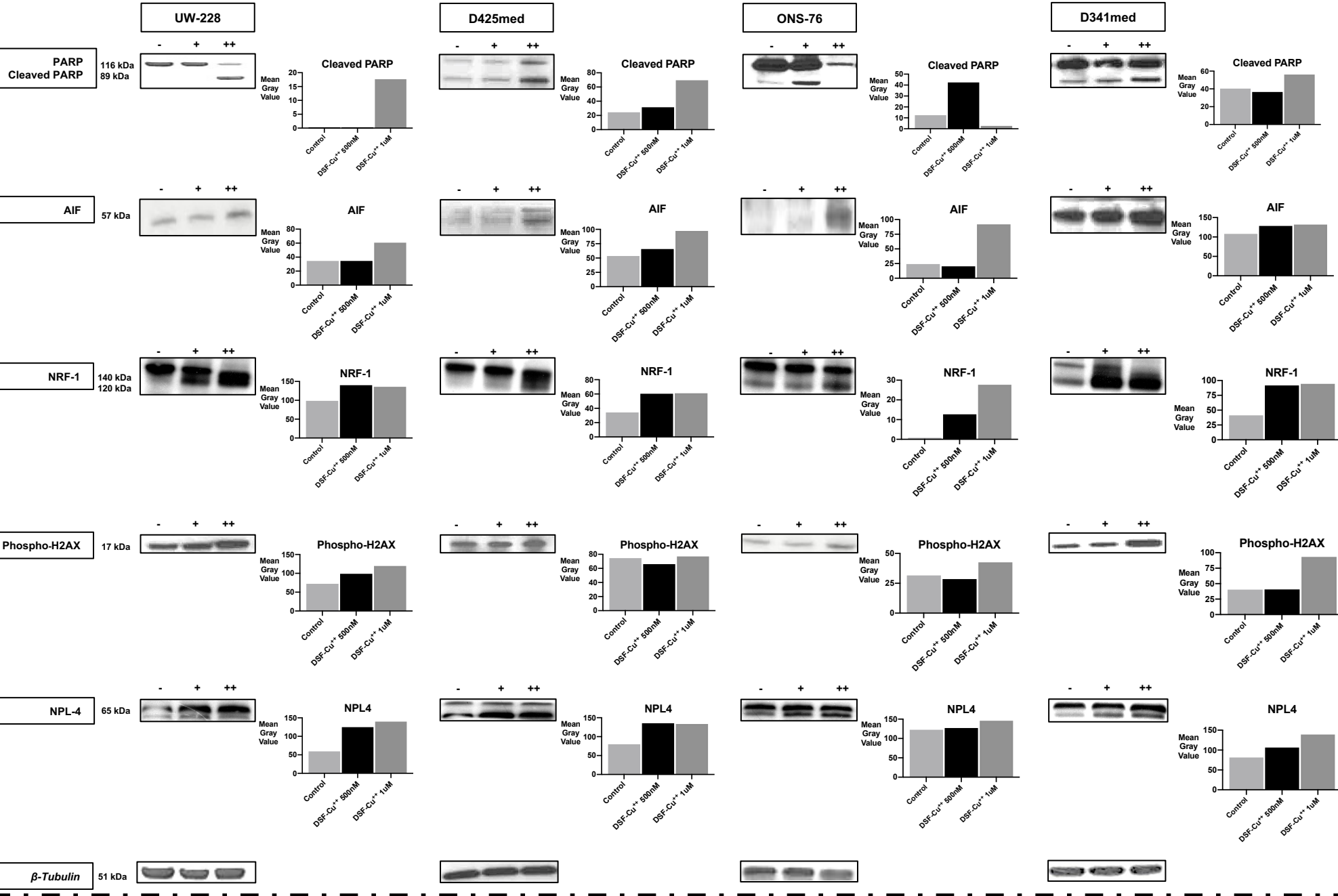

Panel B

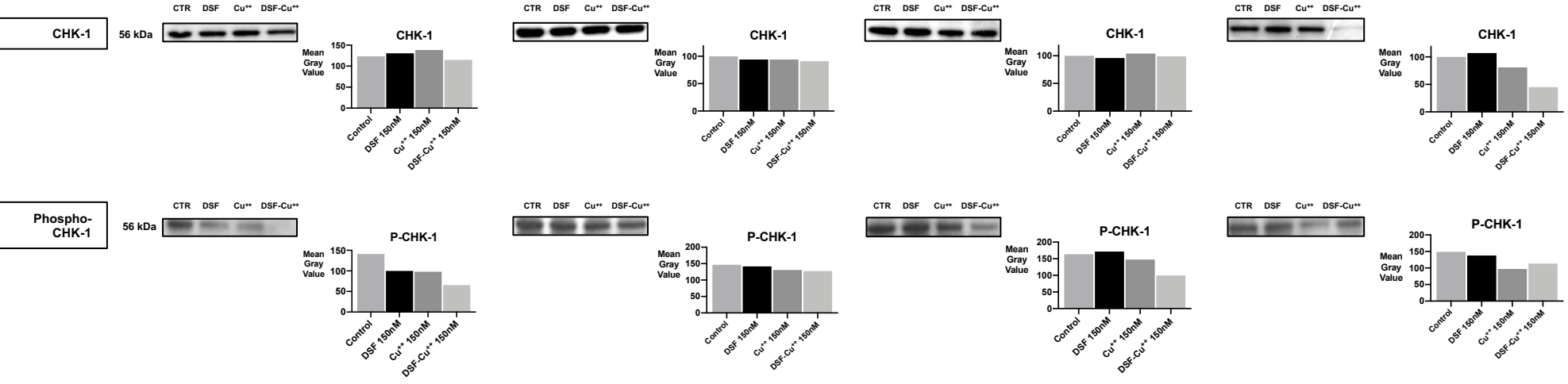

Panel C

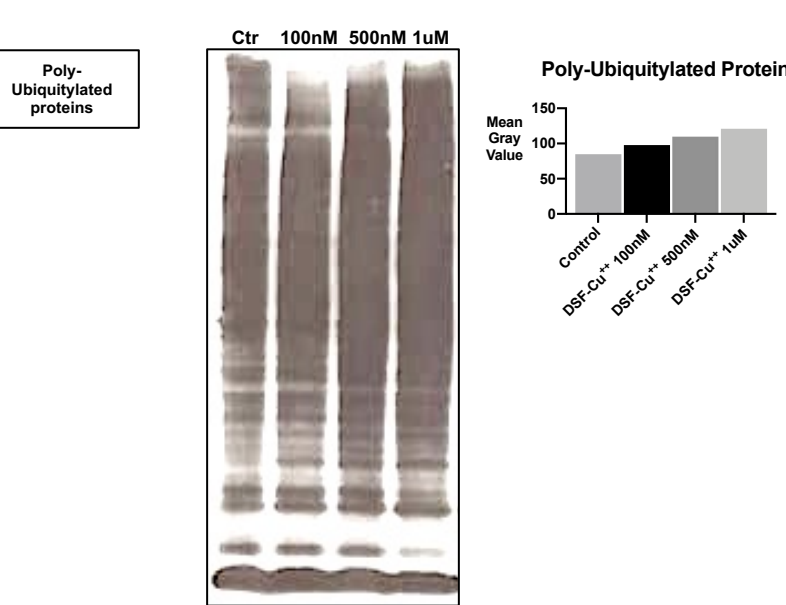

Supplement: S8 Fig — Western blot films presented in Fig 3 obtained from in vitro assays were quantified with ImageJ for band intensity and plotted with GraphPad Prism. (PDF) [file pone.0251957.s008.pdf]

Panel D

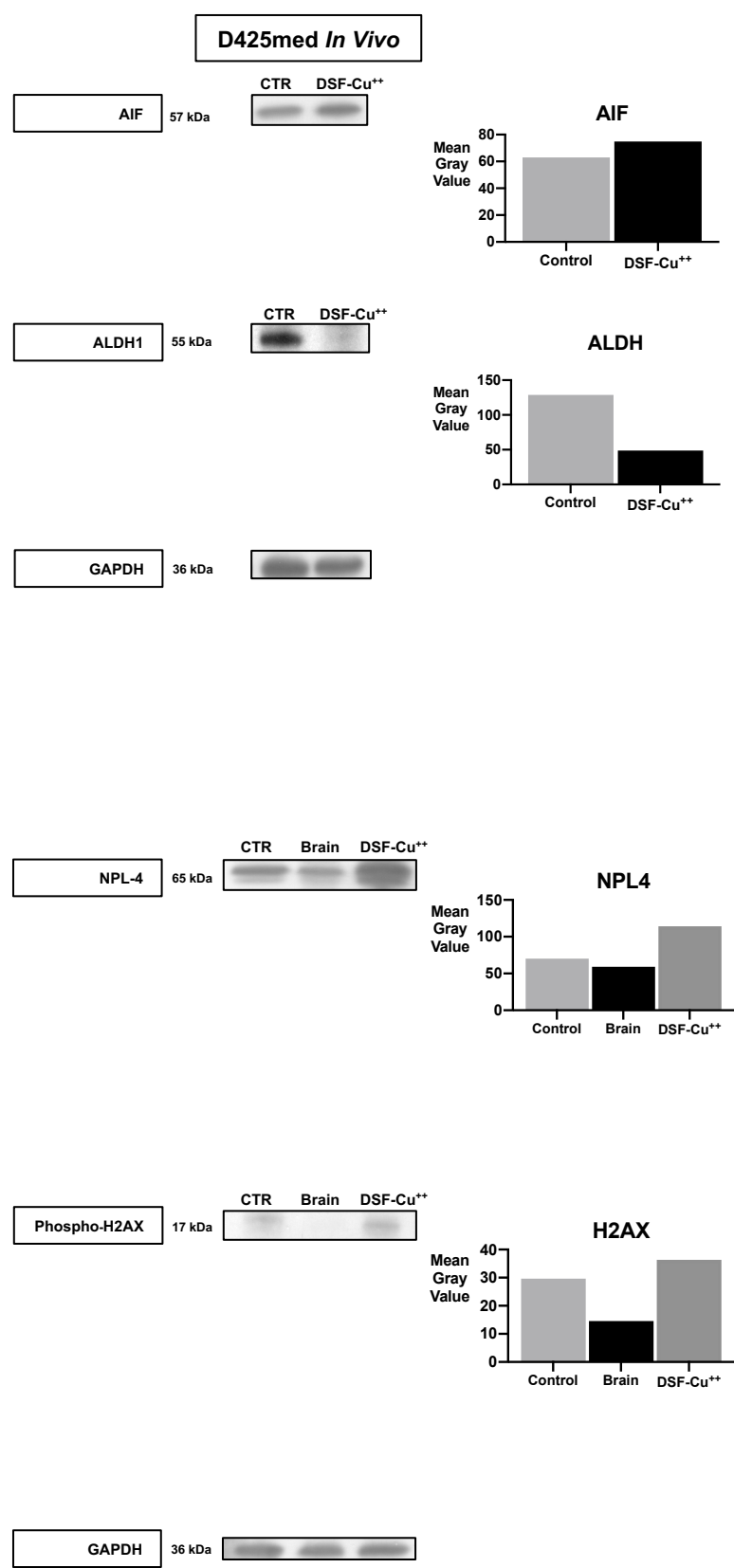

Supplement: S9 Fig — Western blot films presented in Fig 3 obtained from in vivo experiments were quantified with ImageJ for band intensity and plotted with GraphPad Prism. (PDF) [file pone.0251957.s009.pdf]

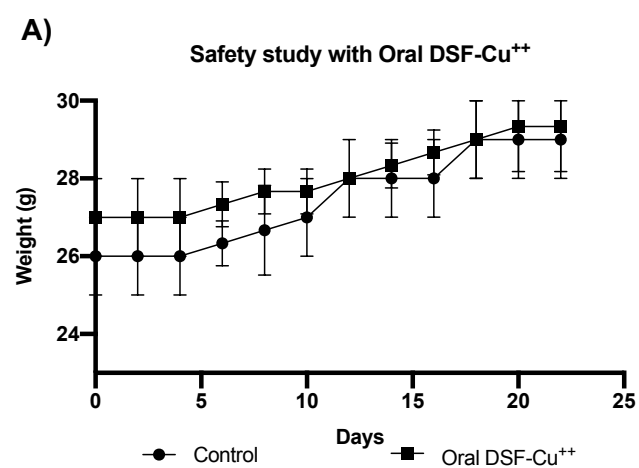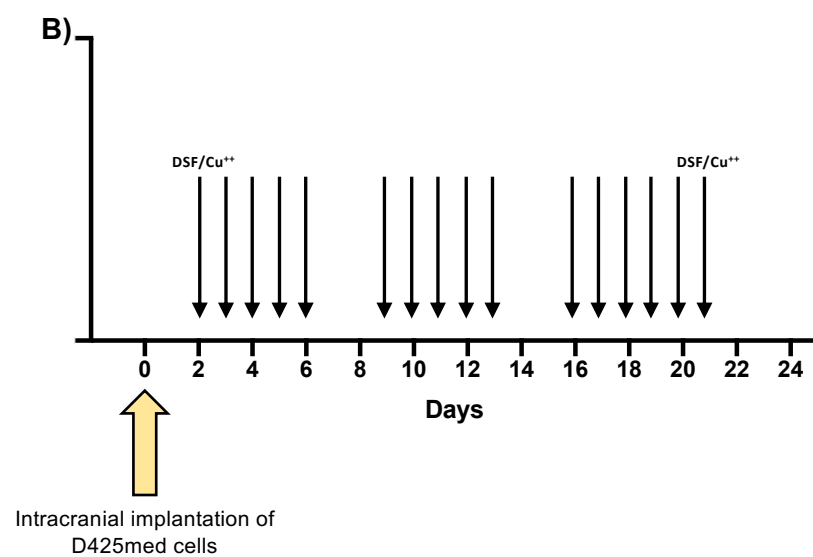

Supplement: S10 Fig — (PDF) [file pone.0251957.s010.pdf]
