# Supplementary material for: Disulfiram and copper combination therapy targets NPL4, cancer stem cells and extends survival in a medulloblastoma model
Source: PLoS One. 2021 Nov 3;16(11):e0251957. doi: 10.1371/journal.pone.0251957 (PMC8565761; doi:10.1371/journal.pone.0251957)

**Supplementary Figure 3**

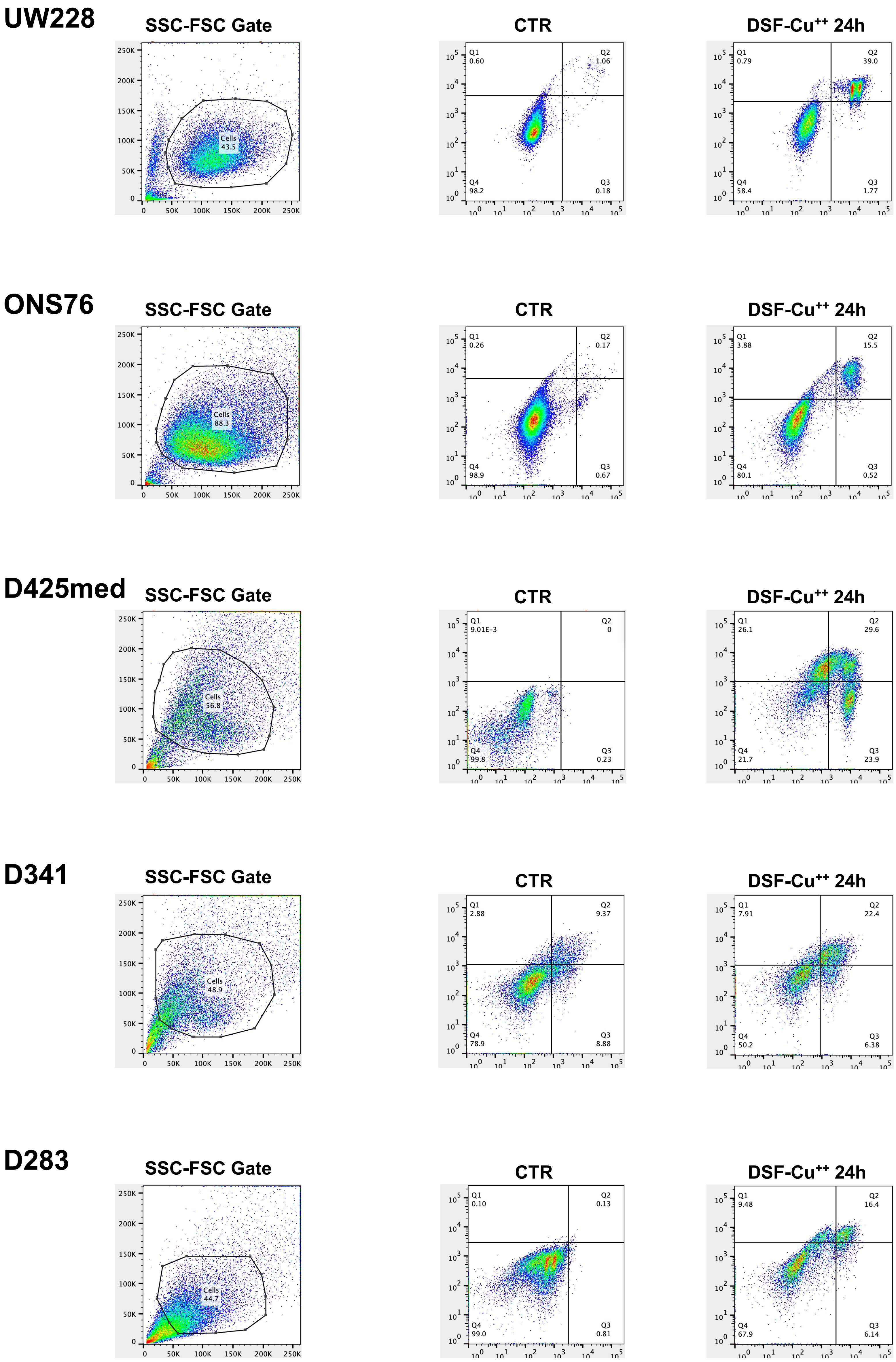

Supplement: S3 Fig — AnnV/PI staining showed a significant increase in AnnV+-PI- and AnnV+-PI+ cells after 12-24h and 24-48h, respectively. DSF-Cu++ was used at a concentration of 150nM in all cell lines. SSC/FSC gating is shown for each cell line, as well as AnnV/PI gating in control and DSF-Cu++-treated samples. (PDF) [file pone.0251957.s003.pdf]
