# Supplementary material for: Disulfiram and copper combination therapy targets NPL4, cancer stem cells and extends survival in a medulloblastoma model
Source: PLoS One. 2021 Nov 3;16(11):e0251957. doi: 10.1371/journal.pone.0251957 (PMC8565761; doi:10.1371/journal.pone.0251957)
Supplement: S4 Fig — SSC/FSC and Ki67 gating is shown for all five cell lines (control and DSF-Cu++-treated 150 nM for 24h). Statistical analysis of cells treated with DSF-Cu++ 150nM and 300nM for 24h and 48h is shown on the right. (PDF) [file pone.0251957.s004.pdf]

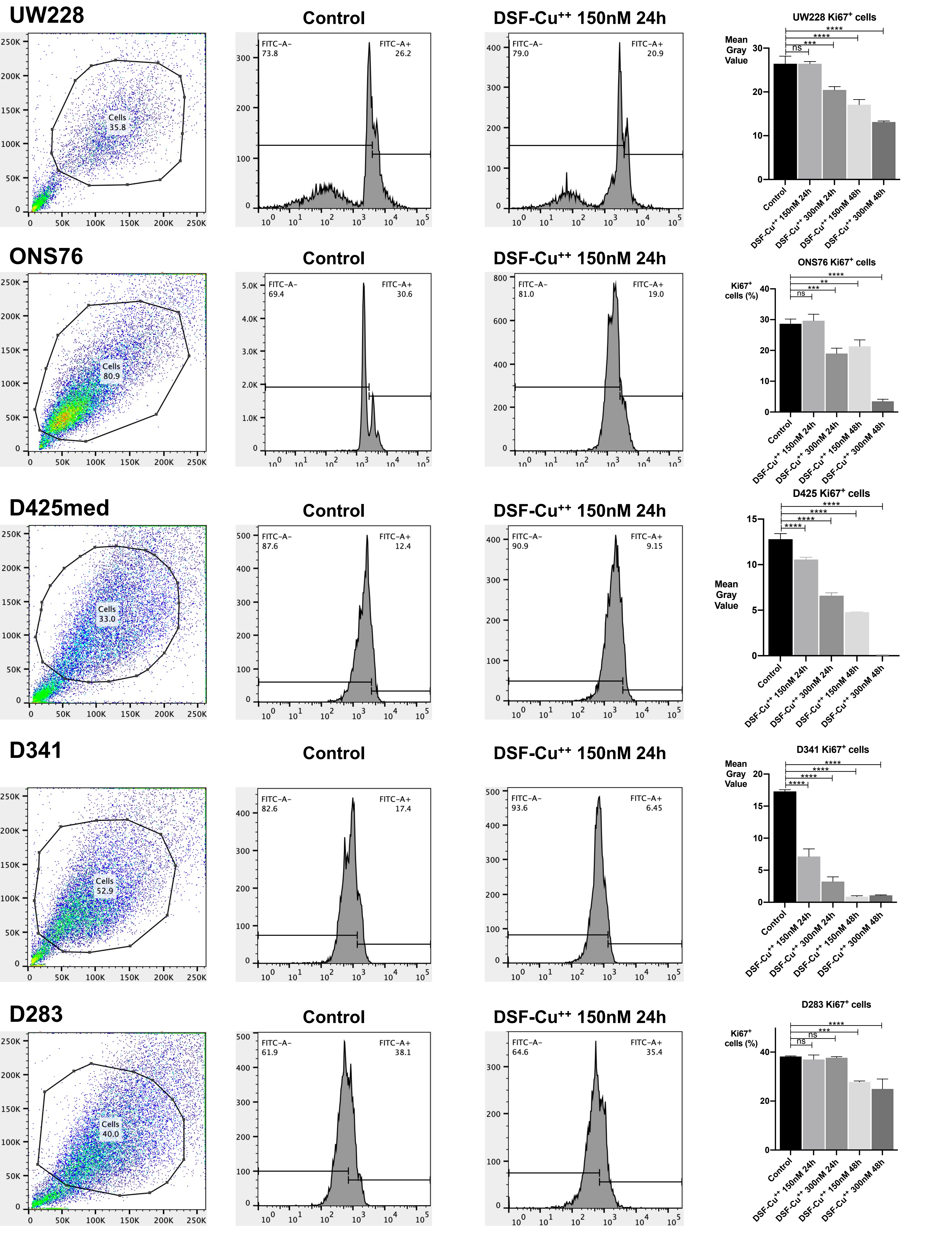

Supplementary Figure 4
