# Supplementary material for: Disulfiram and copper combination therapy targets NPL4, cancer stem cells and extends survival in a medulloblastoma model
Source: PLoS One. 2021 Nov 3;16(11):e0251957. doi: 10.1371/journal.pone.0251957 (PMC8565761; doi:10.1371/journal.pone.0251957)
Supplement: S7 Fig — A) ONS76, D425med and D341 cells showed nuclear and cytoplasmic clustering of NPL4, expression of nuclear foci of H2AX and AIF translocation after treatment with DSF-Cu++. Signal intensity was analyzed with ImageJ and plotted with GraphPad Prism on three replicates per group. Legend;—control, + 150 nM for 24h. (PDF) [file pone.0251957.s007.pdf]

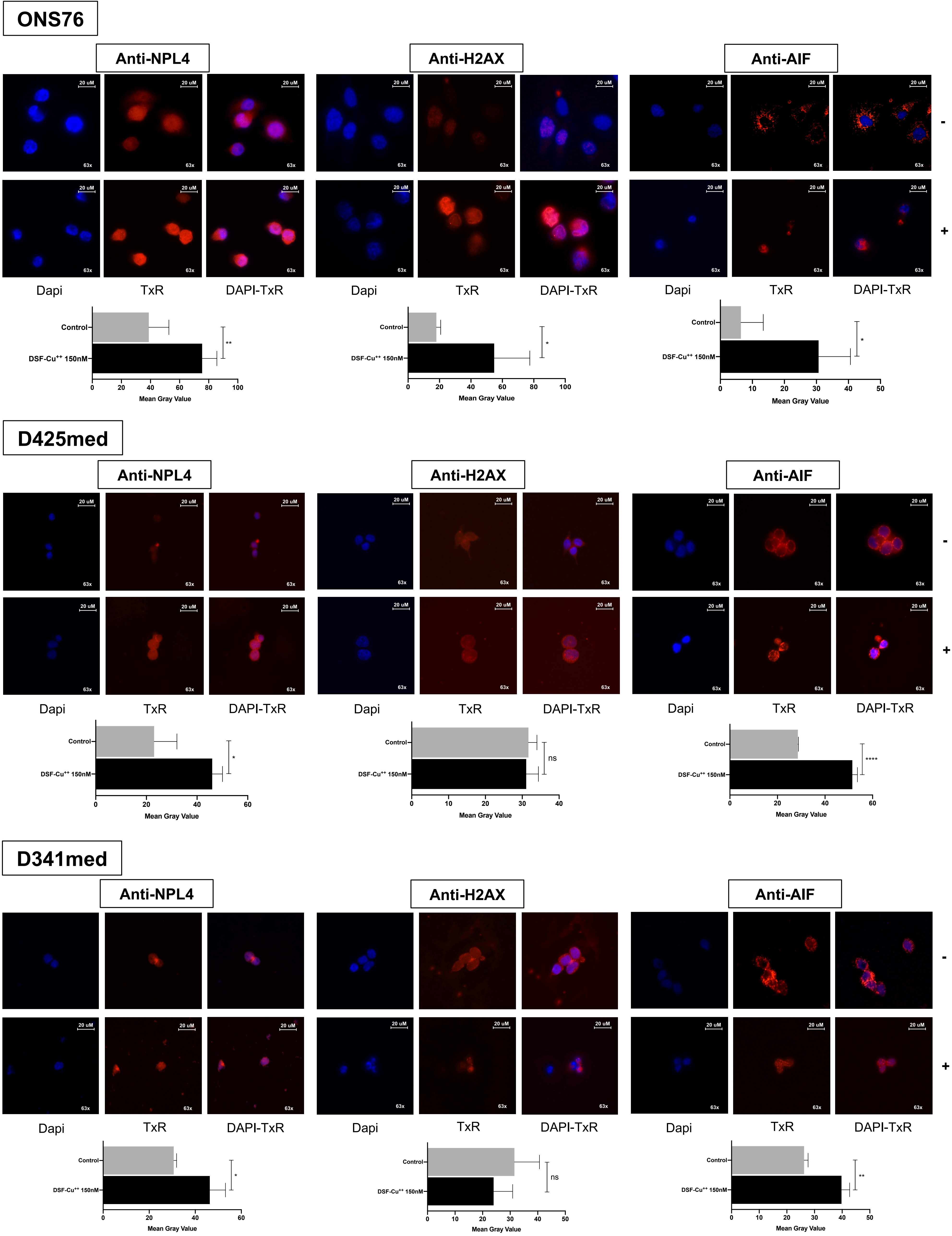

Supplementary Figure 7
